# Correction: Renal cancer survival in clear cell renal cancer compared to other types of tumor histology: A population-based cohort study

**DOI:** 10.1371/journal.pone.0342010

**Published:** 2026-01-29

**Authors:** Teesi Sepp, Antti Poyhonen, Anneli Uusküla, Andres Kotsar, Thea Veitonmäki, Teuvo L. J. Tammela, Aleksei Baburin, Teemu J. Murtola

The Funding statement for this article is incorrect. The correct Funding statement is as follows: This work was supported by the Estonian Research Council grant number PRG2218. The funders had no role in study design, data collection and analysis, decision to publish, or preparation of the manuscript.
